# The Morita-Baylis-Hillman Reaction: Insights into Asymmetry and Reaction Mechanisms by Electrospray Ionization Mass Spectrometry

**DOI:** 10.3390/molecules14103989

**Published:** 2009-10-12

**Authors:** Verónica Carrasco-Sanchez, Mario J. Simirgiotis, Leonardo S. Santos

**Affiliations:** 1 Laboratory of Asymmetric Synthesis, Chemistry Institute of Natural Resources, Universidad de Talca, P.O. Box 747, Talca, Chile; 2 Department of Forestry, University of Concepción, J. A. Coloma 201, Los Angeles, Chile; E-Mail: msimirgiotis@udec.cl (M.J.S.)

**Keywords:** Morita-Baylis-Hillman, reaction mechanism, ESI-MS, online screening

## Abstract

This short review presents new insights on the mechanism and online monitoring using electrospray ionization tandem mass spectrometry (ESI–MS/MS) of Morita–Baylis–Hillman (MBH) reactions. MBH reactions are versatile carbon-carbon organocatalyzed bond forming reactions, making them environmentally friendly due to general organocatalysts employed. The organocatalyst behavior, which controls the transition state and thus the enantioselectivities in the obtained products, is very important in the performance of asymmetric MBH transformations. Some recent techniques and advances in asymmetric transformations are reviwed, as well as online reaction monitoring and analysis of the reaction intermediates. The mechanism accepted nowadays is also review through the insights gained from the use of ESI–MS/MS techniques.

## 1. Introduction

Many efforts have been made in Organic Chemistry in the search for synthetic routes using efficient and clean carbon-carbon bond forming reactions. The most important carbon-carbon reactions discovered so far are the Grignard, Wittig, aldol, Friedel–Crafts, Diels–Alder, Heck, Stille, Suzuki and the Morita–Baylis–Hillman reactions. Of those important C-C bond forming reactions, the Baylis-Hillman or Morita–Baylis–Hillman (MBH) reaction is a very useful and versatile one, which combines aldol and Michael reactions in a single step, providing organocatalyzed methylene--hydroxy carbonyl or -methylene--aminocarbonyl compounds. These compounds are very useful multifunctional building blocks in the synthesis of medicinally relevant pharmaceutical compounds [1,2] and natural products [3]. The interest in this atom-economical reaction has been growing, and good reviews regarding the development and progress using this reaction have been published [4,5,6,7]. The scope of this short review is to discuss some important features involved for the understanding and further developments in this interesting synthetic reaction. Herein, some important selected reactions, recent developments and important insights on the mechanism based on MBH reactions online monitored by electrospray ionization tandem mass spectrometry (ESI–MS) are also reviewed.

## 2. Mechanism. Earlier Work

**Scheme 1:**
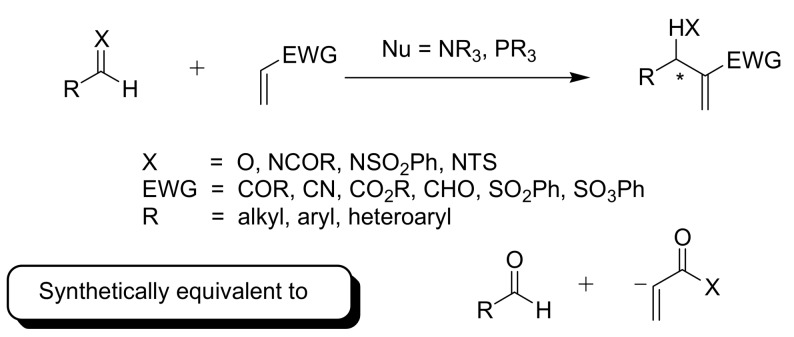
General scope of the Morita-Baylis-Hillman reaction.

This carbon–carbon bond forming reaction is a three component reaction which involves the coupling of an electron deficient olefin acting as Michael acceptor with an electrophile in the presence of a strong Lewis base (a tertiary amine: generally diazabicyclo[2.2.2]octane, DBU or phosphines), as depicted in Scheme 1 [6].

**Scheme 2:**
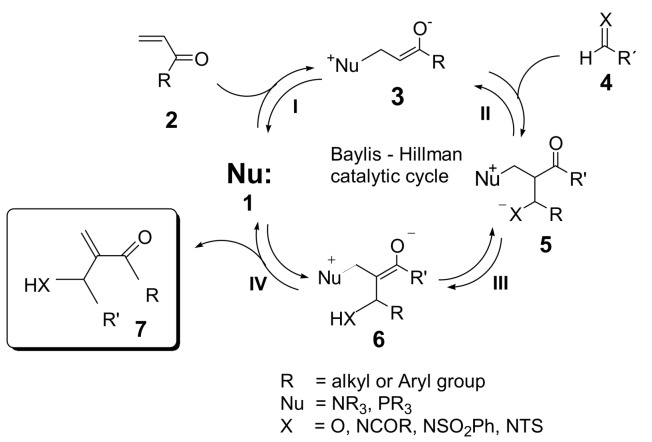
Currently accepted catalytic cycle for the MBH reaction.

The mechanism initially proposed by Hill and Isaacs [8] and refined by others [9,10] was based on the rate, pressure dependence and kinetic isotope effect (KIE) data of the reaction and involves three steps, consisting of a sequence of Michael addition, aldol reaction, and elimination (Scheme 2). The conjugate Michael addition of a Lewis basic catalyst **1** to the α,β-unsaturated carbonyl system of compound **2** initiates the catalytic cycle (step I). This reaction produces the zwitterionic intermediate **3**, which has enhanced nucleophilic reactivity at C-2 through the action of the nucleophile. The aldehyde **4** then reacts with this intermediate (step II), leading to formation of the dipolar intermediate **5**, which after prototropic rearrangement forms intermediate **6** (step III). This intermediate suffers either E2 or E1cb elimination in the presence of a Lewis base to produce the target Baylis–Hillman adduct **7** (step IV).

Hill and Isaac concluded that no α-proton cleavage occurred in a series of MBH reactions [8], thus indicating step II (addition of enolate to the aldehyde) to be the rate determining one, based on a kinetic isotope effect of 1.03 ± 1 for the α-position of acrylonitrile.

## 3. Recent Analysis of MBH Reaction Mechanism

**Figure 1:**
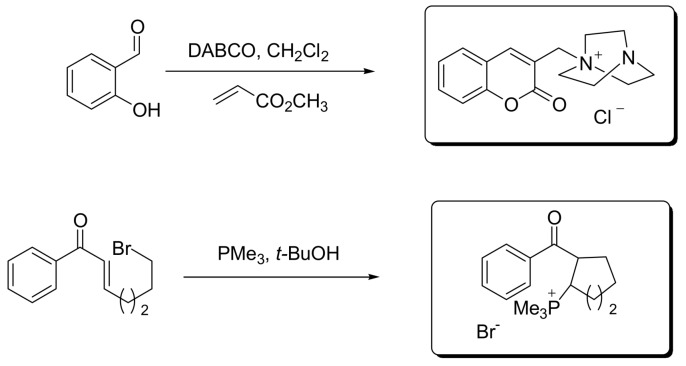
Isolated MBH intermediates 6 as proposed by Isaac.

The proposed key intermediates **6** present in the catalytic cycle, as described in Scheme 2, were isolated and characterized by NMR and X-ray crystallography experiments in the form of a coumarin [11] and phosphonium salts [12] (Figure 1). The data support the mechanism previously proposed by Isaac [8].

**Scheme 3:**
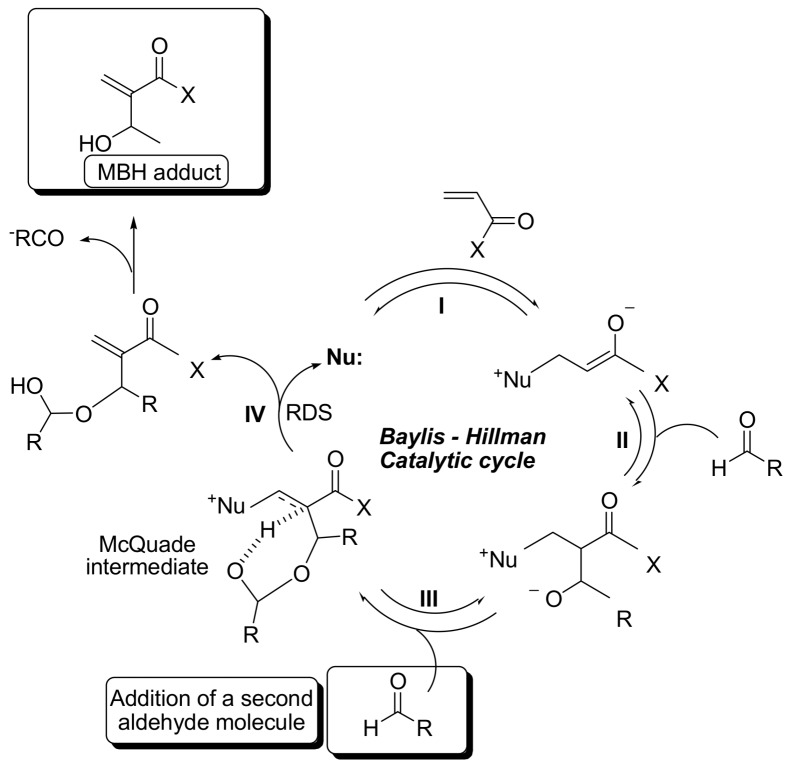
Mechanism involving a hemiacetal intermediate as proposed by McQuade [13,14].

According to that mechanistic proposal, it can be deduced that the reaction could be accelerated using protic additives that could activate the aldehyde. McQuade *et al.* have determined that the Baylis–Hillman rate-determining step is second order in aldehyde and first order in DABCO and acrylate, showing significant kinetic isotopic effect (KIE, k_H_/k_D_ = 5.2 ± 0.6 in DMSO). Interestingly, regardless of the solvent used (DMF, MeCN, THF, CHCl_3_), the KIE were found to be greater than 2, indicating the relevance of proton abstraction on the rate-determining step (RDS). They proposed a new mechanism involving a hemiacetal intermediate, as depicted in Scheme 3 [13,14].

**Scheme 4:**
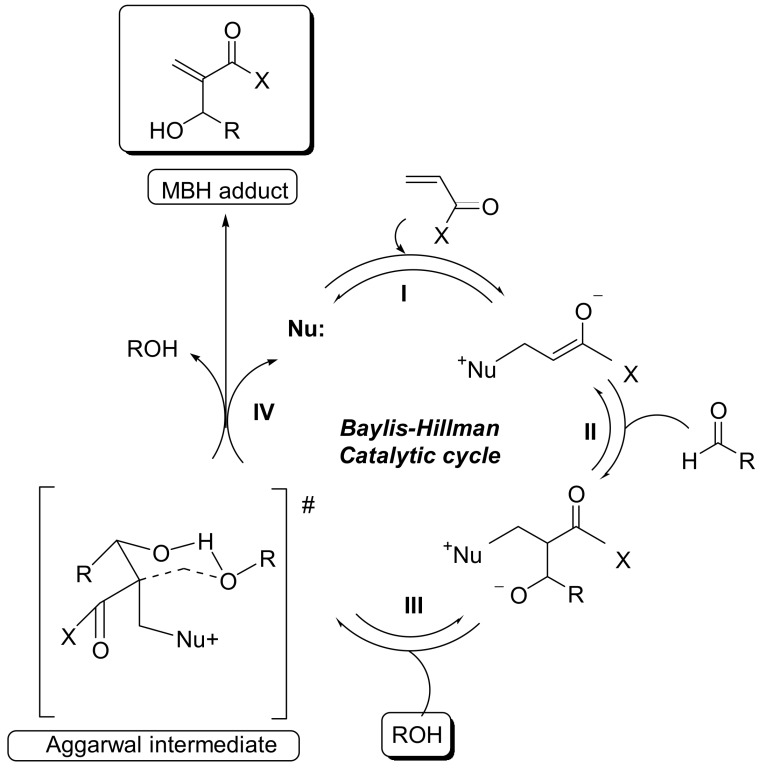
Proposed mechanism involving a six-membered intermediate by Aggarwal [15].

Furthermore, Aggarwal proposed, based on kinetic studies, that the reaction kinetic is second order in relation to the aldehyde, but only at in the initial (≤20% of conversion) stage, then becoming autocatalytic. Aggarwal proposed that the MBH adduct may act as a proton donor and therefore assists the elimination step via a six-membered intermediate [15], as shown in Scheme 4.

Robbiette *et al.* carried out MP2 calculations on a model system focusing on the reaction between methyl acrylate and benzaldehyde catalyzed by a tertiary amine in the absence/presence of methanol [16]. Furthermore, Roy *et al.* used *ab initio* calculations to analyze the rate limiting step between acroleine and formaldehyde catalyzed by trimethyl amine (model system), and between methyl vinyl ketone and benzaldehyde (real system) [17]. As suggested by McQuade, these analyses explained the observed second order kinetics with respect to aldehyde concentration in the absence of protic solvent. However, Aggarwal *et al.* suggested that the presence of alcohol in the reaction media was acting in proton transfer from carbon to oxygen. Moreover, the intramolecular proton transfer transition state was further stabilized. Both theoretical studies confirmed that hydrogen transfer was the rate limiting step, and C-C bond forming should not be rate-limiting, except perhaps for some aliphatic aldehydes or imines. These results suggested that the design of asymmetric versions of MBH requires the stereocontrol of the proton transfer step, in addition to that of the addition to aldehydes [16].

## 4. Reaction Times, Yield and Stereochemistry of MBH Reactions

The main drawbacks of this synthetically important reaction are the slow rate, long reaction times, expensive catalysts, difficult handling and low yields. The most important of these is the long reaction time; it has been shown typically to take days to weeks to obtain acceptable product yields. Another important issue is the stereoselectivity of the reaction. The usefulness of this reaction has encouraged researchers to employ different alternatives to shorten the reaction times, increase the yields and improve the enantioselectivity. Within this scenario, several combinations of the three essential components for MBH reaction, i.e., activated alkene, electrophile, and catalyst as well as the cooperative effect of hydrogen bonding additives such as water, phenols, alcohols, ionic liquids and different reaction conditions such as ultrasound, temperature, and high pressure have been investigated during the last decades [4,5].

### 4.1. Stereochemistry of Michael acceptor

**Figure 2:**
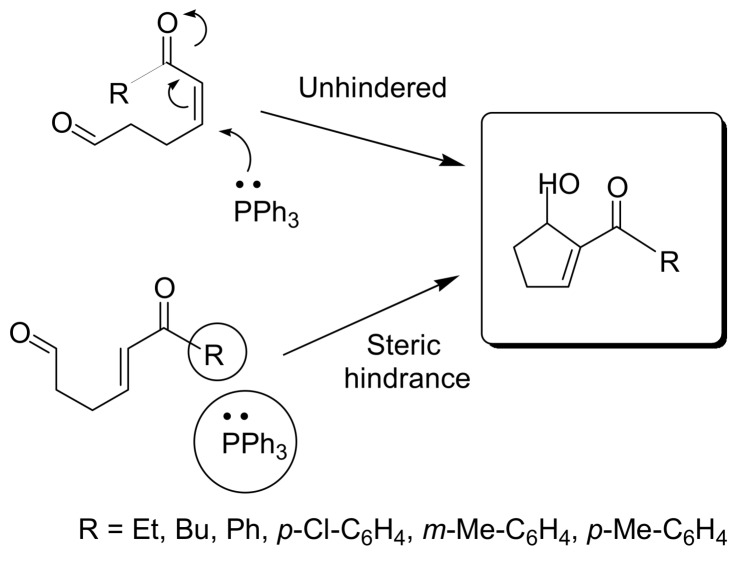
Influence of the stereochemistry of the Michael acceptor in the MBH reaction.

Teng *et al.* showed that differences in the accessibility to the β-position of the Michael acceptors could result in great discrepancies in the yields of the MBH reactions. The group studied the stereochemistry effect of Michael acceptors involved in intramolecular MBH reactions catalyzed by PPh_3_, and using isomerically pure E and Z ω-formyl α,β-unsaturated carbonyl compounds as reaction substrates. In all studied cases, the same product was obtained using both isomers. However, the (*Z*)-alkene afforded 2.5–8.5 times higher yield than the *E* isomer [18]. The authors rationalized the data due to steric hindrance, as depicted in Figure 2.

### 4.2. Studies on the reactivity of the formyl group

**Figure 3:**
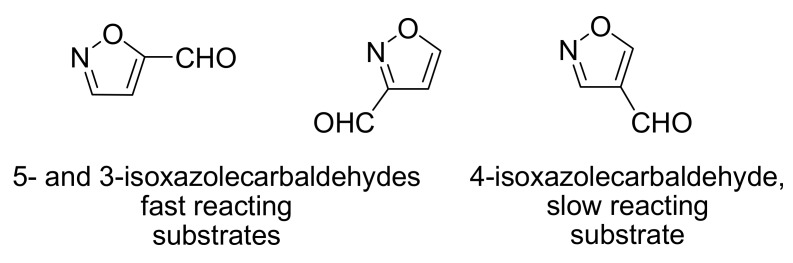
Isoxazolecarbaldehydes used in MBH reactions.

**Scheme 5:**
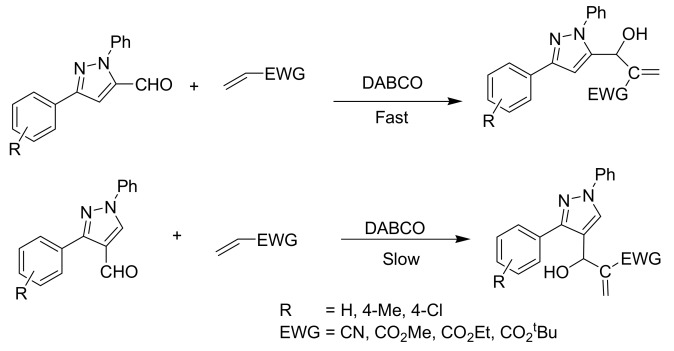
MBH reaction of pyrazolecarbaldehydes.

It is well known that changing from unsubstituted to substituted benzaldehydes has a direct influence on the rate of MBH reactions and affects the formyl group reactivity. Thus, nitro- or trifluoromethyl-substituted benzaldehydes comprise the fastest reacting substrates due to their electron-withdrawing nature, whereas methoxy- or dimethoxybenzaldehydes show the slowest, because of their electron donating character. In heterocyclic systems this is not completely true, and Nag *et al.* studied the reactivity of heterocyclic formyl derivatives involved in MBH reactions. Based on previous results with isoxazolecarbaldehydes (Figure 3), and in order to study the difference in reactivity of formyl groups in heterocyclic systems, they studied substituted pyrazolecarbaldehydes at different positions. Employing DABCO as catalyst, 

Nag found that heterocyclic formyl group presented on the carbon atom adjacent to the heteroatom showed short reaction times (3.5 to 48 h, Scheme 5) for the MBH reaction as compared to other substituted positions (4 to 25 days) [19]. 

**Scheme 6:**
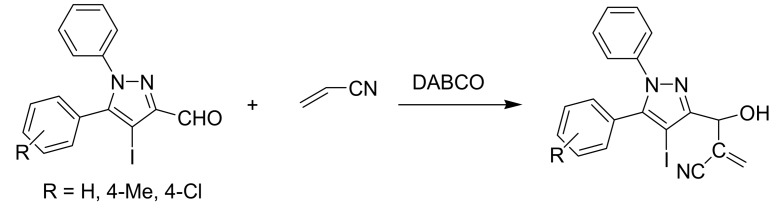
MBH reactions of iodo-pyrazolecarbaldehydes.

It was proposed that the electron pairs of heteroatoms near the formyl group accelerated the base elimination in the final step of the reaction. The presence of halo-substituents in the adjacent carbon to the one bearing the formyl group increased the rate for the same reason (Scheme 6).

### 4.3. Three-component and one-pot MBH reactions

**Scheme 7:**
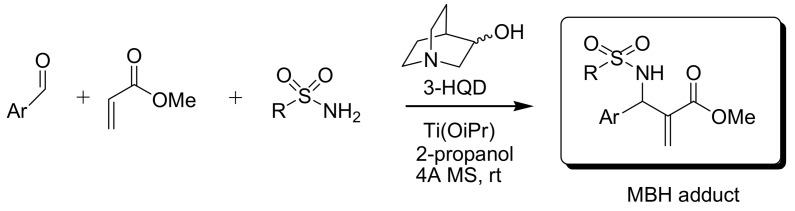
MBH reaction between methyl acrylate and sulfonamides in presence of 3-HQD and titanium isopropoxide.

Adolfsson and coworker employed as catalyst 3-hydroxyquinuclidine (3-HQD, 15 mol%) and titanium isopropoxide (2 mol%) for the reactions of methyl acrylate and sulfonamides in the presence of molecular sieves and 2-propanol at room temperature. Using this three-component one pot system yields and chemoselectivity of the aza-adducts was improved. Furthermore, yields over 94% and selectivity reaching 99% were obtained in reaction times around 6 h (Scheme 7) [20].

**Scheme 8:**
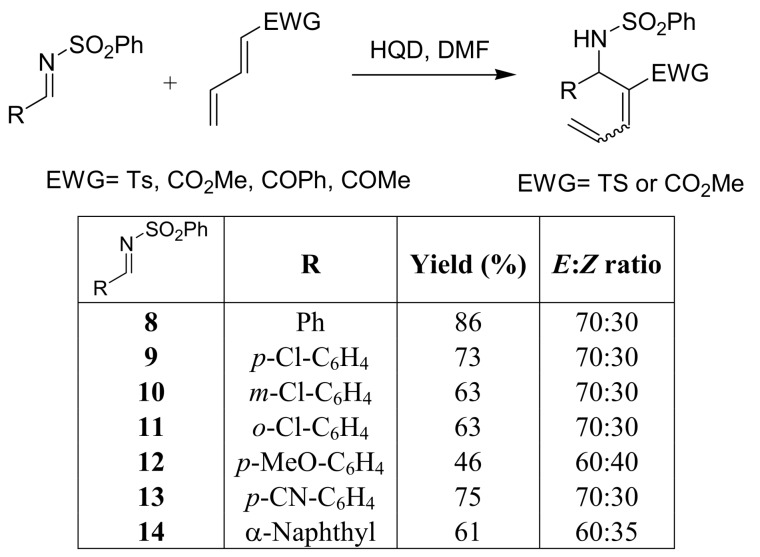
Aza-MBH reaction of substituted imines with several activated conjugated dienes.

**Scheme 9:**
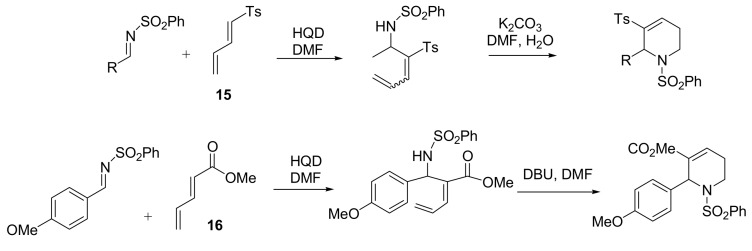
Aza-MBH reaction of substituted imines with compounds 15 and 16 followed by cyclization.

In 2006, Sorbetti and coworkers reported the aza-MBH reaction of substituted *N*-(phenylsulfonyl)-aldimines with several activated conjugated dienes. The reaction afforded the corresponding adducts in the presence of 3-hydroxyquinuclidine (3-HQD, Scheme 8) as highly functionalized allylicamine compounds. Furthermore, it was seen that the *E* isomers of the products obtained from the dienoate ester **15** and the dienyl sulfone **16** underwent one pot facile intramolecular conjugate additions to give the corresponding piperidine compounds [21], as depicted in Scheme 8 and Scheme 9.

**Scheme 10:**
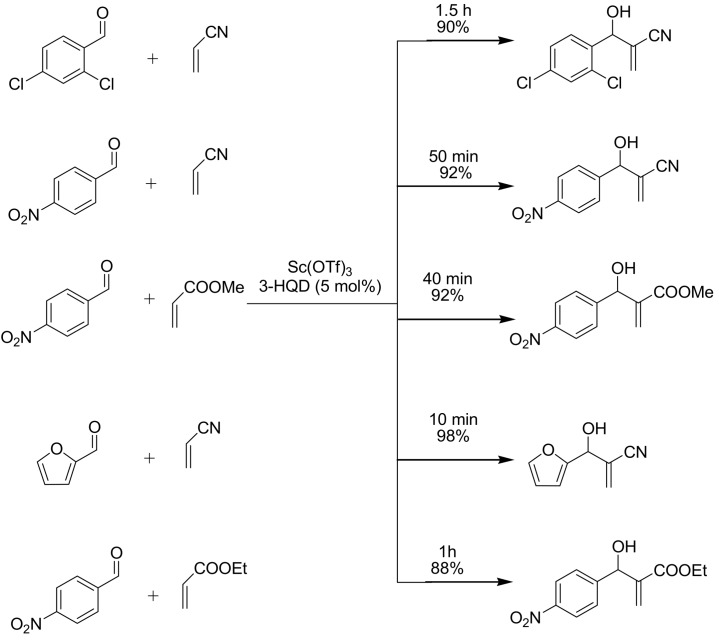
MBH reaction between several aromatic aldehydes and activated alkenes catalyzed by Sc(OTf)_3_ and 3-HQD.

Shang and coworkers reported a combination of Sc(OTf)_3_ and 3-hydroxyquinuclidine (3-HQD) as catalytic system for the MBH reaction between several aromatic aldehydes and activated alkenes. The protocol showed high catalytic activity with good yields and reaction times as short as 10 min. Substrates with acceptor groups (Scheme 10) gave good rate improvement, whereas presenting donor groups showed lower rate accelerations. However, no reaction was observed employing acrylamide [22].

### 4.4. Catalytic asymmetric induction for MBH reactions

MBH reactions are organocatalyzed reactions, which make them environmentally friendly when organocatalysts used are designed and accessible to organic chemists, and very important in the performance of asymmetric transformations and control of the transition state and thus the chirality of the product obtained in the reaction with high enantioselectivity. The diversity of chiral catalysts tested included Lewis acids and Lewis bases, Bronsted acids, thioureas, bulky ammonium salts, ionic liquids and phosphines [4]. Of those organocatalyst employed for asymmetric MBH reactions, bifunctional organocatalysts via hydrogen bonding has been of great interest in the last years, and specially for the aza-MBH reaction, the compounds isocupreidine [20], phosphinyl BINOLs [23], and (*S*)-3-(*N*-isopropyl-*N*-3-pyridinylaminomethyl) BINOL [24] are the most efficient bifunctional catalysts employed so far. Several asymmetric MBH reactions were reported, and two reviews regarding asymmetric MBH reactions were recently published [4,7], so we will mention some important and recent advances in this field. 

#### 4.4.1. Asymmetric induction using chiral (thio)ureas

**Figure 4:**
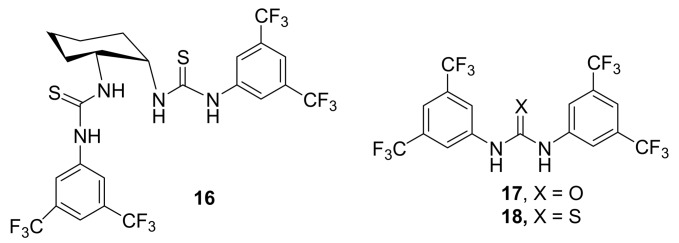
Urea and (thio)urea type organocatalysts used for MBH reaction between cyclohexenone and benzaldehyde.

**Scheme 11:**
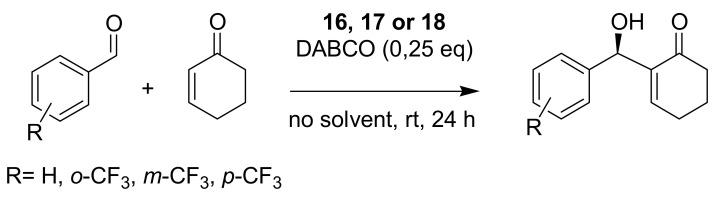
MBH reaction catalyzed by urea or (thio)urea catalysts.

**Scheme 12:**
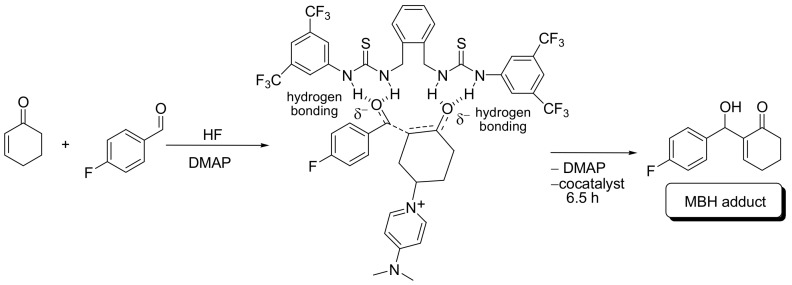
Reaction between cyclohexenone and 4-fluorobenzaldehyde catalyzed by DMAP and **19**.

**Figure 5:**
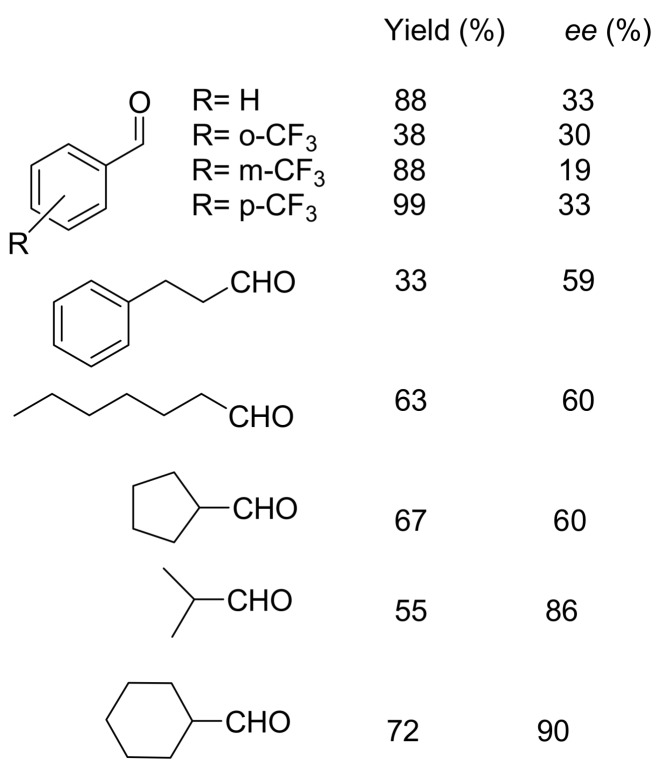
Aldehydes used in MBH reactions catalyzed by *bis*(thio)urea catalyst **16**.

**Figure 6:**
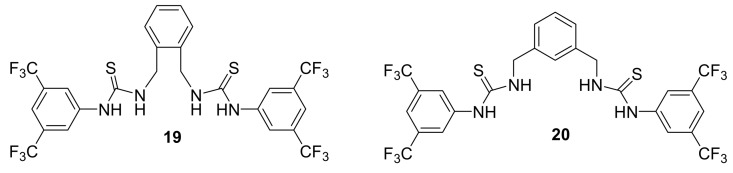
*Bis*(thio)urea type organocatalysts for the MBH reaction between cyclohexenone and 4-fluorobenzaldehyde catalyzed by DMAP.

An important contribution was the work of Hatakeyama *et al.*, that found that β-isocupreidine (β-ICD) was an efficient catalyst for the MBH reaction of acrylate with various aromatic and aliphatic aldehydes affording mostly *R* allylic alcohols (99% *ee*) [25]. This attractive solution stimulated more studies on organocatalysis in MBH reactions. Initially, the efforts concentrated on increasing MBH reaction rates, but soon after asymmetric approaches using chiral ureas as catalyst have became the main targets. Nagasawa *et al.* [26] performed the MBH reaction between cyclohexenone and benzaldehydes catalyzed by DABCO and cocatalyzed with urea or (thio)urea type organocatalysts (**17** or **18**, Figure 4, Scheme 11), obtaining yields of 52-60% after 24 h. However, the chiral bis-thiourea organocatalyst **16** provided good yields (72%) and a 33% *ee*. After improvement of the reaction conditions employing cyclohexenone, several aldehydes (Figure 5), cocatalyst **16**, and DMAP at 5 °C, the allylic alcohols were obtained in 72 h with enantiomeric excesses up to 90% *ee* for cyclohexanecarboxaldehyde. Recently, Connon *et al.* also described the use of (thio)ureas as efficient organocatalysts for MBH reactions for deactivated aromatic aldehydes with significant increases in rate and yield [27]. Another example is given by Jones *et al*. based on this type of molecules. The group synthesized two *bis*(thio)urea cocatalysts (**19** and **20**, Figure 6) designed by calculations of electronic structure of a key transition state for the reaction between cyclohexenone and 4-fluoro-benzaldehyde catalyzed by DMAP. The condition employed showed that docking the transition state by hydrogen bond mediated recognition of both the nucleophile and the electrophile accelerated the reactions in 4 times [28] (Scheme 12).

#### 4.4.2. Asymmetric induction using biphenols

**Figure 7:**
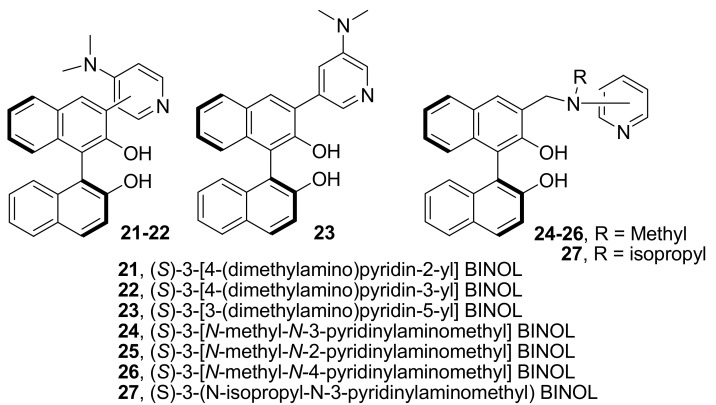
Several Biphenols derivatives used as catalyst for MBH reaction between methyl vinyl ketone and phenyl *N*-tosylimine.

**Scheme 13:**
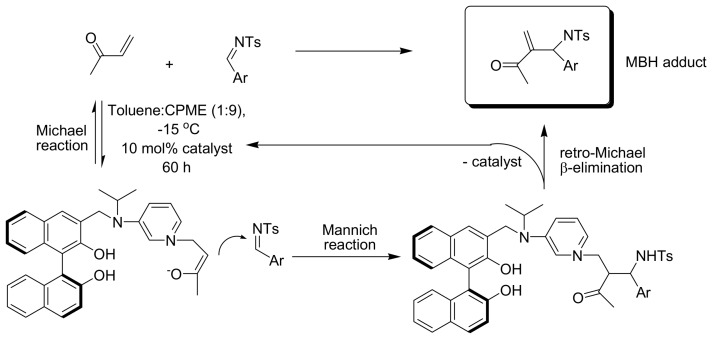
Mechanism of MBH reaction methyl vinyl ketone and phenyl-*N*-tosylimine catalyzed by (*S*)-3-(*N*-Isopropyl-*N*-3-pyridinylaminomethyl)BINOL (**27**).

The MBH reaction between methyl acrylate and imines was first reported by Perlmutter and Teo in 1984 [29], and generated what is called the aza-MBH reaction. The development of asymmetric organocatalysts to carry out efficient aza-MBH reactions has been a challenge in organic synthesis in the last years. Matsui *et al.* performed aza-MBH reaction of methyl vinyl ketone and phenyl *N*-tosyl-imine with several organic catalysts, which had Bronsted acids and Lewis base functionalities. Of several tested BINOLs (Figure 7), they found the compound (*S*)-3-(*N*-isopropyl-*N*-3-pyridinyl-aminomethyl) BINOL **27** to be the most efficient giving 96% yield (60 h) and high enantioselectivity (95% *ee*) [24]. The reaction was shown to be highly influenced by the position of the Lewis base attached to the BINOL structure (see proposed mechanism, Scheme 13). The authors suggested that the acid and base groups cooperated to activate the substrate, and lock the conformation of the organocatalyst, promoting the reaction with high enantioselectivity.

#### 4.4.3. Asymmetric induction using phosphines

**Scheme 14:**
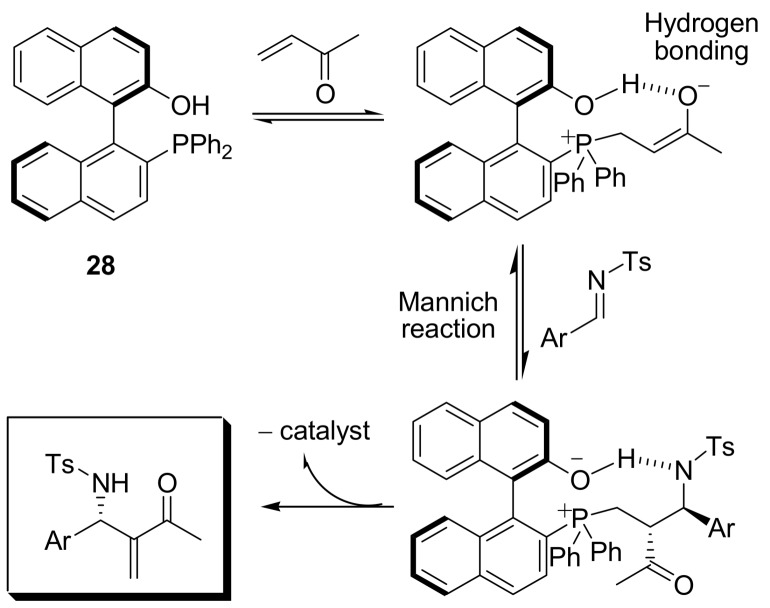
Proposed MBH reaction of methyl vinyl ketone with *N*-tosylimine catalyzed by the diphenylphosphino binaphthalenol **28**.

The search for stereoselectivity in MBH reactions started with Shi that employed chiral phosphines derived from BINAP (**28**) for the reaction of tosylimines with methyl vinyl ketone [23]. The proposed reaction mechanism consisted of stabilization of the starting enolate thorough hydrogen bonding. Then, Mannich reaction of this intermediate with tosylimine and subsequent β-elimination afforded only one of the possibly four diastereomers (Scheme 14).

**Scheme 15:**
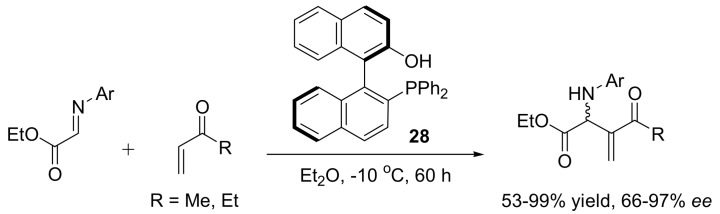
MBH reaction of methyl vinyl and ethyl vinyl ketones with *N*-tosylimines catalyzed by **28**.

**Scheme 16:**
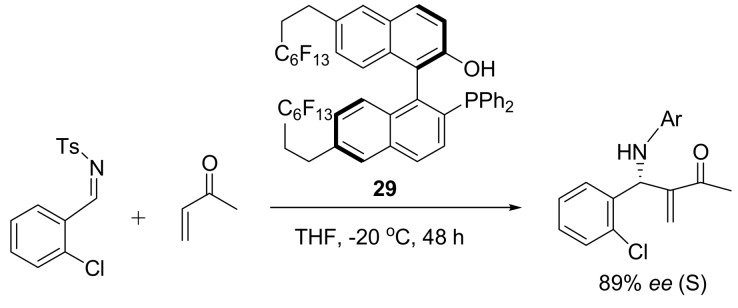
MBH reaction of methyl vinyl ketones with *N*-tosylimines catalyzed by **29**.

This compound, (*R*)-2´-diphenylphosphino-[1,1´-binaphthalene]-2-ol (**28**) also resulted to be one of the most efficient bifunctional phosphine-type chiral catalysts found for the aza-MBH reaction of methyl vinyl and ethyl vinyl ketones with *N*-tosylimines [30] that afforded excellent enantiomeric excess and good yields (Scheme 15). In another work, Shi and coworkers synthesized the catalyst with an additional C_8_F_13_ side-chain (ponytail, compound **29,** Scheme 16). Testing catalyst **29** for MBH reaction, enantiomeric excesses reaching 90% chemical yield 90% and reaction times around 48 h were observed [31].

#### 4.4.4. Asymmetric induction using chiral amines

**Scheme 17:**
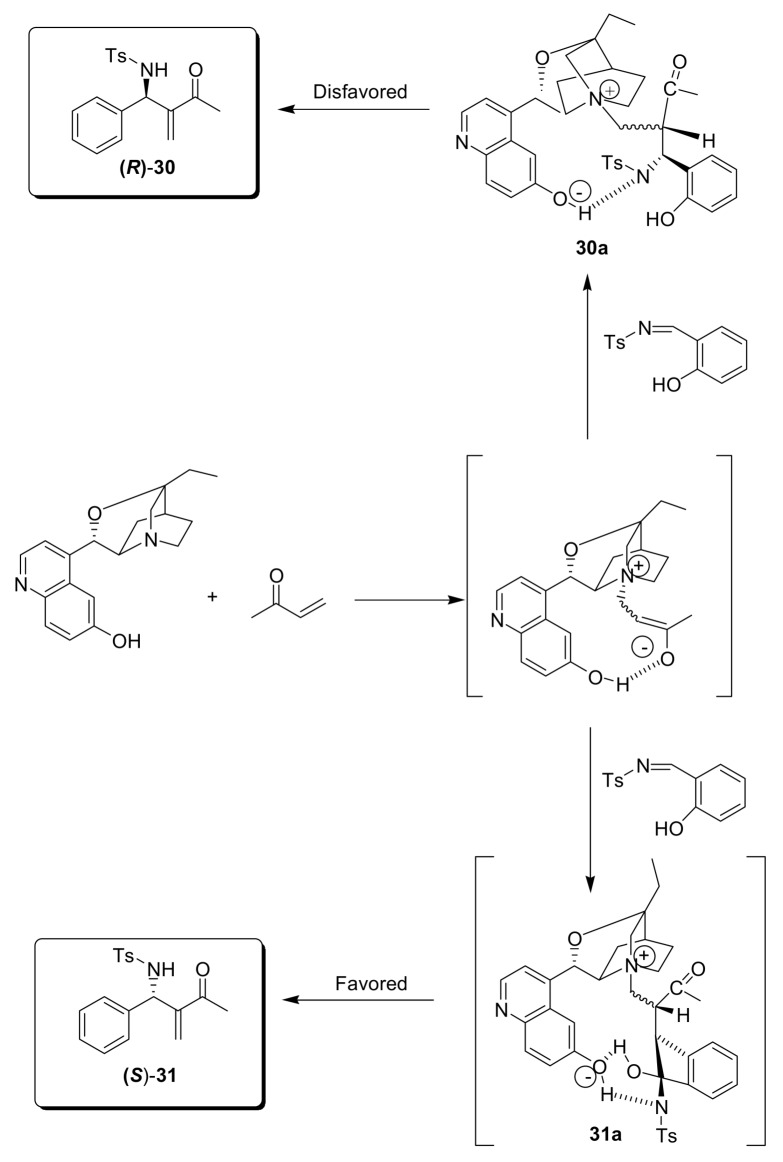
MBH reaction between *N*-tosylsalicylaldehyde and an, α,β-unsaturated ketone catalyzed by isocupreidine.

An excellent example of asymmetric aza-MBH reaction was performed using the quinidine derived base isocupreidine (ICPD, as the catalyst in 10 mol%) in THF at –30 °C. Shi *et al.* reported a highly efficient reaction of *N*-tosylsalicylaldehyde imines with α,β-unsaturated ketones to give the corresponding adducts in good to high yields and excellent enantioselectivity (up to 99% *ee*). It was proposed that intermediate **31a** would be more stable than intermediate **30a**, because the phenol containing aromatic moiety in the chiral pocket could form a strong branched hydrogen bonding system achieving aza-MBH adducts in the *S*-configuration. Shi suggested that in the transition state of intermediate **30a**, the *ortho*-phenol group was located outside of the chiral environment (Scheme 17), thus affording low selectivities [32].

**Scheme 18:**
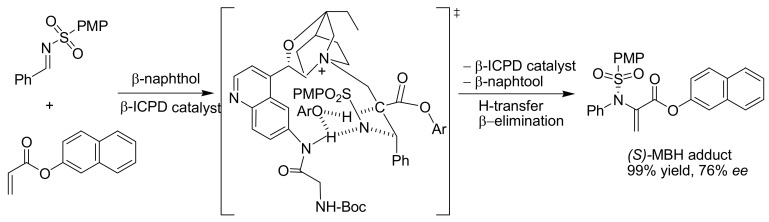
MBH reactions of aromatic imines catalyzed by ICPD derived catalyst and β-naphthol derivative.

An interesting approach was described by Zhu and coworkers that obtained high yield and enantioselectivity through dual catalysis. A combination of ICPD derived bifunctional-catalyst and β-naphthol for the asymmetric aza-MBH reactions of aromatic imines was employed [33]. A cooperative hydrogen bond pairing was proposed to explain the formation of the stabilized intermediate that lead to the MBH *S*-adduct (Scheme 18).

### 4.5. Amino acids used as catalysts for MBH reaction

**Scheme 19:**
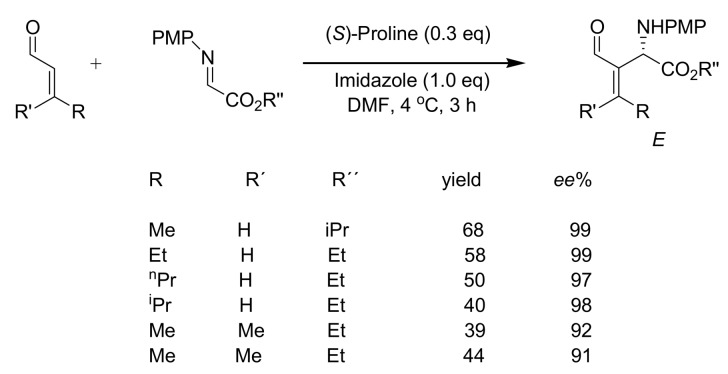
Aza-MBH reactions from **β-substituted-α,β-unsaturated** aldehydes and imino esters catalyzed by proline and/or imidazole.

**Scheme 20:**
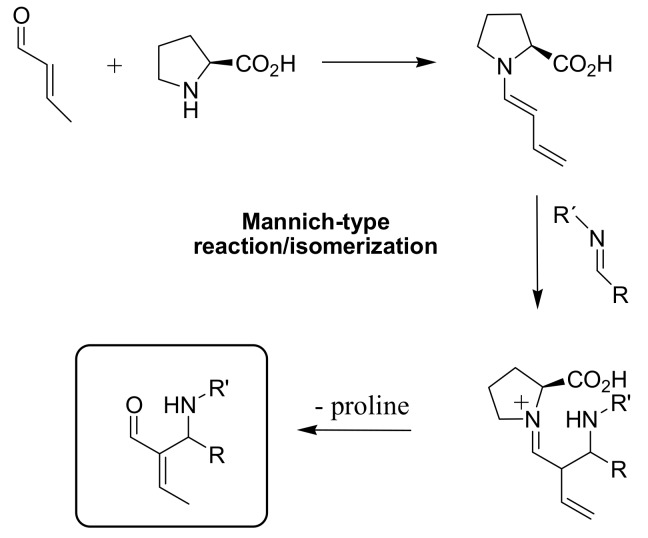
Proposed mechanism for MBH reactions from **β-substituted-α,β-unsaturated** aldehyde and imino esters catalyzed by proline.

Utsumi and coworkers synthesized aza-MBH type compounds with β-substituted enal moieties from β-substituted-α,β-unsaturated aldehydes and *p*-methoxyphenyl protected imino esters (Scheme 19). The reactions were carried out under mild conditions through proline and/or imidazole catalysis, which gave good yields and *ee*% after reaction times of 2 h [34]. The proposed mechanism was a Mannich-type reaction of *in situ* generated enamines of the β-substituted-α,β-unsaturated carbonyl compounds followed by double bond isomerization, as described in Scheme 20.

#### 4.5.1. Polymers as organocatalysts

**Figure 8:**
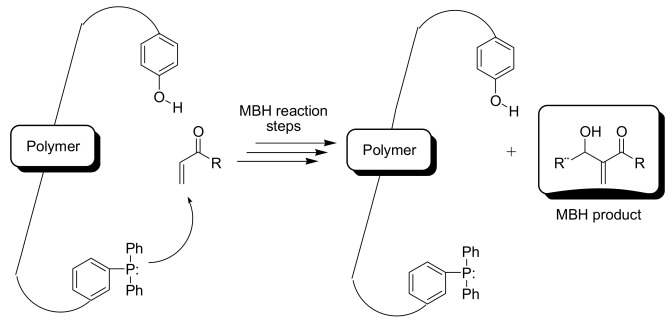
Polymer catalyzed MBH reaction.

Polymers which contained soluble polystyrene-supported triphenylphosphane and 4-dimethyl-aminopyridine with alkyl alcohol (or phenol groups) were also successful as organocatalysts in a range of MBH reactions, as described by Shi and coworkers. The results indicated that hydroxyl groups could participate in the reactions and accelerate product formation, and phenols were more effective as catalyst than alkyl alcohol groups. It was proposed that both functional groups can cooperatively participate in the catalysis of the reactions [35], as described in Figure 8.

### 4.6. Enzymes as catalysts

**Scheme 21:**
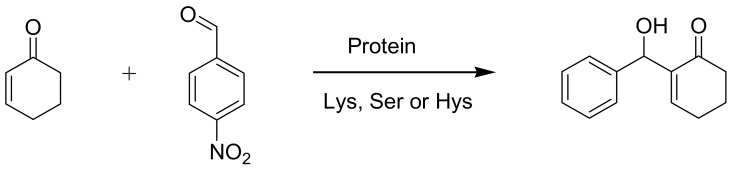
MBH reaction catalyzed by enzymes.

Serum albumins and lipases were also used as catalyst for MBH reaction. Those proteins were able to catalyze the reaction between cyclohexenone and *p*-nitrobenzaldehyde in 35% yield and low enantioselectivities (19%) after 2-5 days in MeCN at 30 ^°^C. It was proposed that the lysine, serine or histidine amino acids of the protein could act as catalytic nucleophiles for this MBH reaction [36], as described in Scheme 21.

### 4.7. Ionic liquids as additives for MBH reaction

**Scheme 22:**
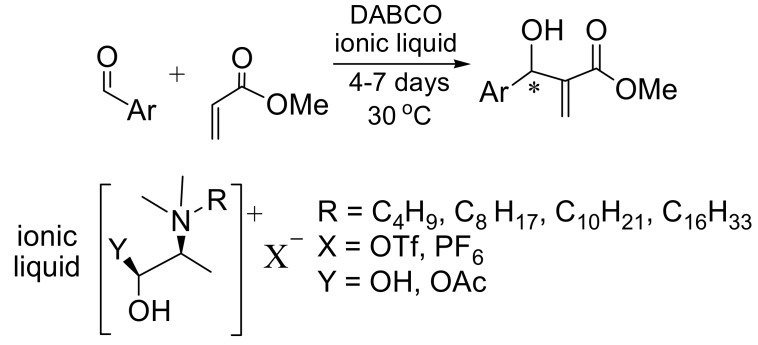
Asymmetric Morita–Baylis–Hillman reaction between benzaldehyde and methyl acrylate using ionic liquids as reaction media.

The use of chiral ionic liquids as reaction media in the asymmetric Morita–Baylis–Hillman reaction between benzaldehyde and methyl acrylate catalyzed by DABCO was reported for the first time by Loupy and coworkers [37]. It gave around 45–76% yields and 20–44% enantiomeric excesses using benzaldehyde, methyl acrylate and DABCO in a 1:1:1 ratio with the addition of 0.5 to 3 equivalents of *N*-alkyl-*N*-methylphedrinium salts (Scheme 22).

**Figure 9:**
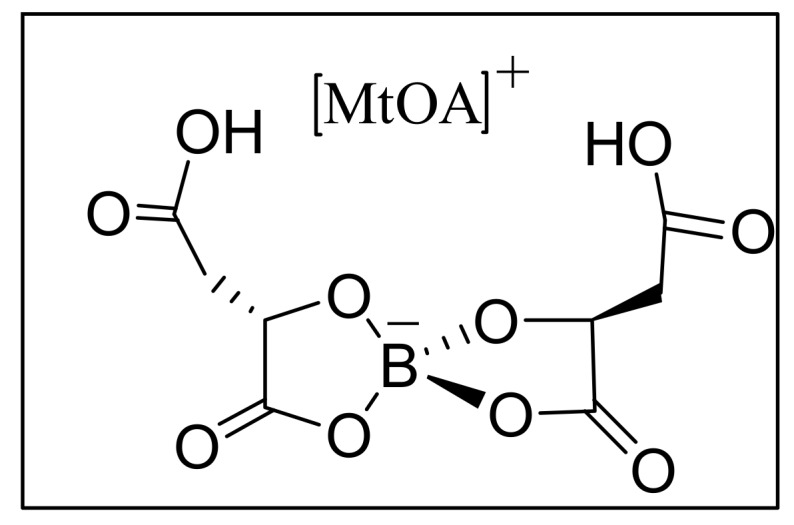
Methyltrioctylammonium dimalatoborate, an efficient ionic liquid developed for chiral induction of MBH reaction between methyl vinyl ketone and *N*-(4-bromobenzylidene)-4-toluenesulfonamide.

Another example of chiral induction using an ionic liquid as solvent was performed for the aza-Baylis–Hillman reaction between methyl vinyl ketone and *N*-(4-bromobenzylidene)-4-toluene-sulfonamide using PPh_3_ as the nucleophilic catalyst. In a series of four independent experiments using various batches of methyltrioctylammonium dimalatoborate (Figure 9), conversions ranging between 34% and 39% and enantioselectivity varying from 71–84% *ee* were achieved [38]. 

**Scheme 23:**
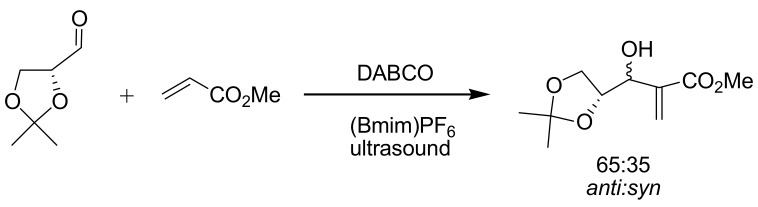
Isopropylidene D-glyceraldehyde and methyl acrylate catalyzed by DABCO and the ionic liquid (Bmim).PF_6_ as additive.

Coelho and coworkers studied a series of MBH reactions between isopropylidene D-glyceraldehyde and methyl acrylate catalyzed by DABCO. It was found that the addition of the imidazolic ionic liquid (Bmim).PF_6_ (1 equiv) increased the yield up to 95% (7% yield without ionic liquids). Furthermore, the use of ultrasound increased the yields up to 82% and decreased reaction times to 30 min at 0 °C. However, the diastereoselectivity was low (65:35 *anti*:*syn* in all the cases) [39], as depicted in Scheme 23.

### 4.8. Acceleration of MBH reaction through mechanochemistry

**Figure 10:**
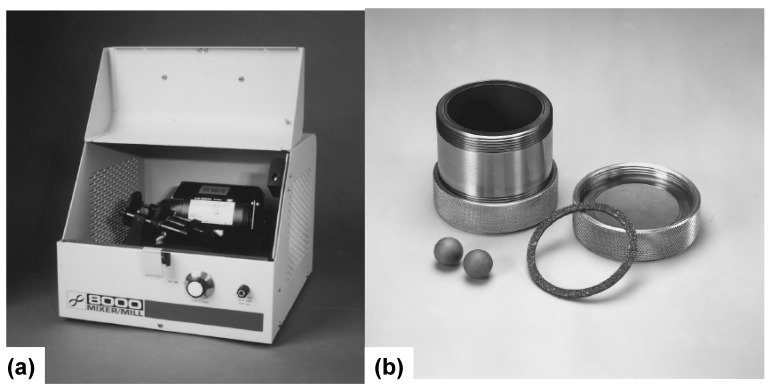
(a) SPEX 8000 mixer/mill in the assembled condition. (b) Tungsten carbide vial set consisting of the vial, lid, gasket, and balls. Courtesy of SPEX CertiPrep, Metuchen, NJ, USA. Reproduced with permission from reference [40].

**Scheme 24:**
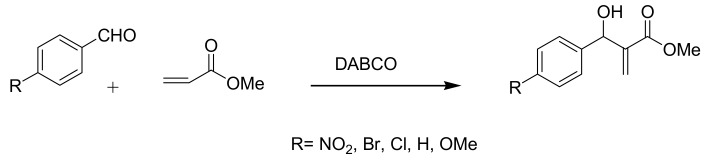
MBH reaction obtained by high speed ball milling.

Mack and coworkers introduced a novel technique of high speed ball milling (HSBM, Figure 10), and performed several reactions between aryl aldehydes and methyl acrylates catalyzed by DABCO to give MBH adducts in reaction times ranging from 30 min to 1 h (Scheme 24). HSBM was employed as an alternative solvent-free method, and consisted of a ball bearing that was placed inside a vessel and shaken at high speeds. The high speed attained by the ball-bearing has enough force to make an amorphous mixture of the reagents facilitating chemical reactions [40]. This method has been studied in metal alloying and for the generation of inorganic salts; however, few organic reactions have been studied by this process. The technique applied to MBH reactions represented one of the fastest options producing yields ranging from 28 to 98% under neat conditions. Furthermore, the technique is an example of green chemistry due to no solvents are required [41].

### 4.9. Online MBH reaction

**Scheme 25:**
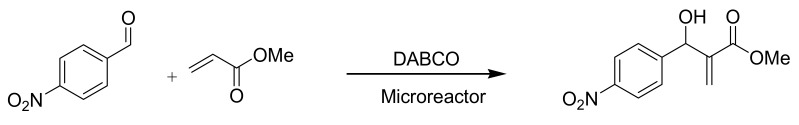
MBH reaction between 4-nitrobenzaldehyde and methyl acrylate obtained online using a microreactor.

The MBH reaction between 4-nitrobenzaldehyde and methyl acrylate was adapted for microreactor conditions (Scheme 25). The reaction could be performed continuously at room temperature in water-dioxane 1:4 ratio (only 2 hours of residence time) catalyzed by DABCO and was approximately 30% faster compared to batch conditions [42].

## 5. Probing the MBH Mechanism by Online ESI–MS(/MS)

Several mechanistic considerations have been proposed for MBH reactions. During the last two decades there has been considerable growth in the development of electrospray ionization mass spectrometry (ESI–MS) as a practical method for studying reaction mechanisms. This tool allows interception and characterization of several key intermediates, either as transient species or as protonated/deprotonated forms of neutral species. Reaction pathways shown by ESI–MS(/MS) have been probed by gas-phase ion/molecule reactions, and expanded mechanisms have been elaborated based on mass spectrometric data. The successful application of ESI–MS in revealing, elucidating, and helping to consolidate proposed mechanisms of organic reactions is emphasized. ESI–MS has been shown to constitute an excellent technique for mechanistic studies, since it transfers ions directly from the solution to the gas phase with efficiency and gentleness, thereby providing proper snapshots of the ion composition of the reaction solutions. This technique has been used extensively to investigate the mechanisms of several classical and organocatalyzed reactions [43,44,45]. For the MBH reaction of acrylates with aldehydes catalyzed by DABCO, several neutral zwitterionic intermediates are involved in the currently accepted mechanism. These neutral species were expected to be in equilibrium with their protonated or cationized forms such as [M + Na]^+^ or [M + K]^+^ in methanolic solutions, and could therefore be detected and analyzed by techniques such as online monitoring electron spray ionization mass and tandem mass spectrometry (ESI-MS/MS). 

### 5.1. Preservation of the charge in the transit of ions from solution to the gas phase using the ESI technique

ESI–MS has previously been used to probe proposed mechanisms based on experimental evidence, and isolation of the different products, thus validating or undermining the empirical proposals. More detailed information about the technique can be found in some excellent reviews [46,47,48] that summarize the current thinking on the various stages of the ESI process [49,50,51]. However, a brief comment on the ESI mechanism must be emphasized.

The electrospray process can be described with relative simplicity. A solution of the analyte is passed through a capillary maintained at high potential. The high voltage generates a mist of highly charged droplets which passes through a potential and pressure gradients towards the analyzer portion of the mass spectrometer. During that transition, the droplets reduce their size by evaporation of the solvent and by droplet subdivision resulting from the coulombic repulsions caused by the high charge density achieved in the shrinkage. As final result, the ions become completely desolvated [52].

The charge state of the isolated ions is expected to closely reflect the charge state in solution (multiply charged species are sometimes observed due to ion/molecule reactions in the interface), since the transfer of ions to the gas phase is not an energetic process – the desolvation is indeed a process that effectively cools the ion [53]. Therefore, it can be assumed that the ESI involves only the stepwise disruption of non-covalent interactions, principally the removal of molecules of salvation, and interception of this process may allow the preservation of relatively strong non-covalent interactions of analytical significance [51]. For example, in a detailed study by Kebarle and Ho, the transfer to the gas phase of different ions dissolved in a wide variety of solvents was possible by ESI. It included singly and multiply charged inorganic ions, e.g., alkali, alkaline earths, and transition metals, organometallic species, and singly and multiply protonated or deprotonated organic compounds, e.g., amines, peptides, proteins, carboxylic acids, and nucleic acids. The experimental information available so far on the preservation of the charge is not clarifying [54]. Single-electron transfer can occur during the electrospray process, because the ESI capillary may act as an electrolytic half-cell when extreme conditions are employed [55]. It is quite common to find a few reports concerning redox reactions in ferrocene derivatives [56], metalloporphyrins [57], metal complexes of amino acids [58], and some organic molecules. Some neutral organic molecules that posse no sites of protonation/deprotonation have also been analyzed using unconventional conditions for ESI technique [59,60,61,62]. In all these examples high ion-source voltages or modifications in the configuration of equipments were carried out to achieve the electrolytic-cell like ESI-MS.

There are some documented cases where the charge state is changed after the desolvation process, like the case of multiply charged inorganic and organic ions, which are sometimes not seen in their expected charge state. The change occurs mainly for ions with the charge localized on one single atom or small group of them, e.g., M^z+^ with z > 1; SO_4_^2–^, PO_4_^3–^[63] When the multiple charge is confined to such a small volume, the high coulombic repulsions present forces the ion to undergo a charge reduction by intra-cluster proton transfer reaction in case of protic solvents, or by charge separation between the metal center and the bonded solvent molecules when aprotic solvents are used. Such reduction processes are promoted by collisions with residual gas molecules in the source interface region [64]. The interception of multiply charged naked ions can be achieved when the charge is stabilized by solvent molecules or ligands. 

The ESI technique is not only qualitative, but the intensity of the detected gas phase ions and the corresponding concentrations of these ions in the electrosprayed solution are related. However, in cases where the solution contains compounds able to react with these ions, the intensities may change drastically and much more complex relationships may prevail. The coordination properties of supramolecular compounds may also differ between the solution and the gas phase. It is well-known that polydentate ligands capable of forming stable solution complexes with transition metal ions favor the transfer of the metal into the gas phase by stabilizing it [65]. Using salts of double charged ions (M^II^X_2_) as an example, ESI of [M^II^L + X_2_] complexes may afford singly [M^II^L + X]^+^ or doubly [M^II^L]^2+^ charged ions by losing one or two counter-ions (X^–^) respectively. In some cases, it is possible to observe ions in the ESI mass spectrum corresponding to [M^II^L – H]^+^, due to the elimination of one counter ion plus a subsequent expulsion of the counter anion acid (HX). The proton is therefore provided by the ligand. Meanwhile in all these cases the oxidation state of the metal center does not change, the coordination number can indeed change, as Vachet and coworkers suggested. For a number of Ni^II^ and Cu^II^ complexes, the coordination numbers differ on going from solution to gas phase [66,67,68,69].

All these cases suggest the necessity to perform a rigorous analysis to prove unambiguously that the species detected by ESI are the ones prevailing in solution, and more importantly to confirm that they are indeed reactive intermediates on the reaction path. An outstanding methodology is to isolate in the gas phase the species assumed to participate in the reaction mechanism and perform ion/molecule reactions with the substrate of the reaction solution. This methodology is very powerful to discard side-products and to assure the reliability of the analysis. Another important method is the study of well-known reactions and compares the data obtained by ESI–MS with other spectroscopic techniques.

#### 5.1.1. Developing methods to study reaction mechanisms

##### 5.1.1.1. Monitoring methods

There are two different possibilities for study a reaction using API methods, *i.e.*, off-line and online screenings. Early investigations using API techniques were usually performed off-line [70,71]. A sequence of events in off-line study of a reaction in solution by mass spectrometry could be: (i) investigate the specific reaction conditions by mixing the reagents for the detection of the different intermediates; (ii) and then determine the solution composition over time when the reactants are progressively transformed into products [72,73]. This latest operation can be accomplished by direct screening by MS of the reaction intermediates during pre-defined intervals and characterization by MS/MS, always if there is a reasonable concentration of them in solution, and these species are not degraded within few seconds/minutes. The overall time resolution has to match the rate of the process to yield the desired information. However, there are some inherent limitations to this approach. Transient species cannot be therefore analyzed with this feature due to their short lifetime residence in solution. 

##### 5.1.1.2. Off-line monitoring

A sequence of events in the study of a reaction in solution by mass spectrometry could be to investigate the specific reaction conditions by mixing the reagents for the detection of the different intermediates, and then determine the solution composition over time when the reactants are progressively transformed into products. This latest operation can be accomplished by direct screening by MS of the reaction intermediates during pre-defined intervals and characterization by MS/MS, always if there is a reasonable concentration of them in solution, and these species are not degraded within few minutes. The overall time resolution has to match the rate of the process to yield the desired information. It is determined by the interval elapsed between consecutive sampling operations and is a direct function of the time used to secure and quench each aliquot in off-line methods. Transient species can not be therefore analyzed with this feature due to their short lifetime residence in solution.

Hinderling and Chen [74,75] used the off-line monitoring technique in developing a mass spectrometric assay of polymerization catalysts for combinational screening. A homogeneous Brookhart polymerization catalyzed by a Pd(II) diimine complex using various ligands was quenched and then studied by ESI–MS. The mass spectrometric method required mg quantities of catalyst, took place in only few minutes, and was suitable for both pooled and parallel screens of catalyst libraries. This intriguing method of high-throughput screening of homogeneous catalyst by electrospray ionization tandem mass spectrometry was recently reviewed [45].

##### 5.1.1.3. Online monitoring

In a second scenario, the kinetic and mechanistic informations about the reactions in solution can be studied using reactors coupled to the ion-source. The first online mass spectrometric investigation in electrochemical reactions using thermospray ionization was reported in 1986 [76]. It proved the potential-dependent formation of dimers and trimers in the electrooxidation of *N*,*N*-dimethylaniline in an aqueous solution. Another example of online investigations is the reaction of ferric bleomycin and iodosylbenzene that was given by Sam and coworkers [77] in 1995, where a low dead volume mixing tee directly attached to the spray source was used. This feature provides mass-specific characterization of stable products and reactive intermediates with lifetimes down to the millisecond time regime, and subtle changes in the reactional medium can also be observed. The simplest reactor online coupled to the mass spectrometer is the own syringe. It allows screening the reaction in real time, and trapping of transient species. Several groups have developed different devices to study the mechanism in solution of radical initiated, photochemical, electrochemical and organometallic reactions, reducing the transit time of the species during the experiments.

#### 5.1.2. Microreactor

##### 5.1.2.1. Peek mixing tee as microreactor

**Figure 11:**
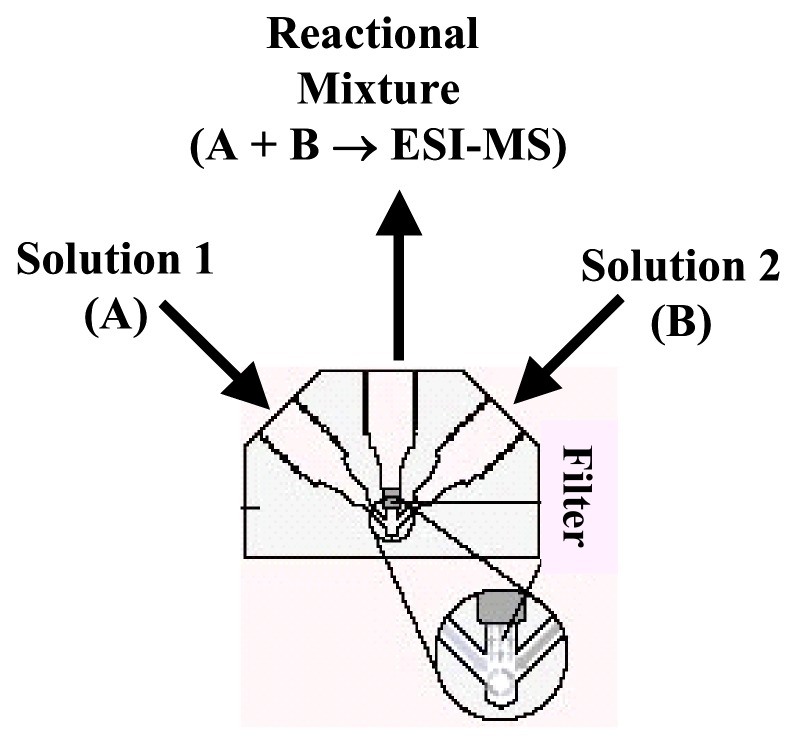
The microreactor allows the effective mixing of the reactants in solution and is coupled directly to the electrospray ion source.

An interesting commercially available PEEK mixing tee (Alltech, USA) can be seen as a useful microreactor [78,79]. It can be directly connected to the ESI spray capillary, and allows covering reaction times from 0.7 to 28 s in a continuous-flow mode. Longer reaction times can easily be covered introducing a fused silica transfer capillary of variable length between the microreactor and the spray capillary. The reaction time is controlled by flow rates from syringes, and by capillary diameters. The chemical reaction in this system takes place by mixing two liquid flows containing the substrate and the reagent in the close proximity of the ionization source. In the moment of mixing both solutions, the reaction is initiated and the mass spectra of the reacting solution under steady state conditions can be acquired (Figure 11).

### 5.2. Detection of MBH cycle intermediates

**Scheme 26:**
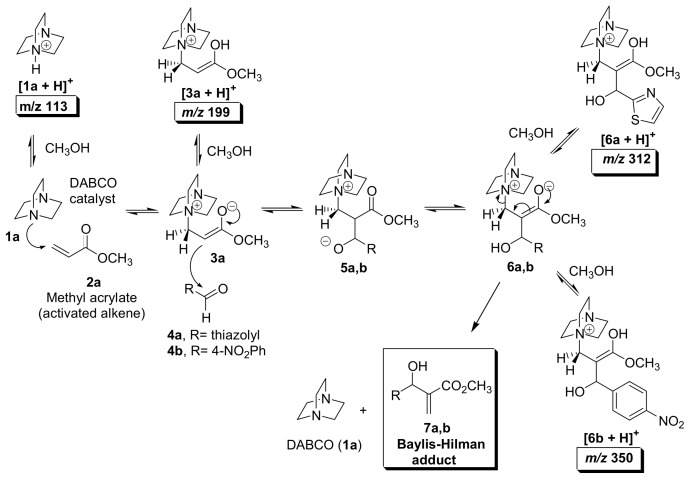
Mechanism of Baylis–Hillman reaction of methyl acrylate (**2a**) and aldehydes **4a,b** catalyzed by DABCO (**1a**). The protonated species expected to be intercepted and structurally characterized by ESI(+)–MS/MS, with their respective *m/z* ratios.

Using ESI–MS in both the positive and negative ion modes, we and coworkers have online monitored MBH reactions. All the proposed intermediates for the catalytic cycle of the MBH reaction (**1-7**, Scheme 2) were for the first time successfully intercepted and structurally characterized. The MBH reactions were performed in a sealed syringe as the reactional medium directly coupled to ion-source [80]. The proposed intermediates for the catalytic cycle of the reaction (Scheme 26) were successfully intercepted and structurally characterized for the first time using ESI–MS and MS/MS. Strong evidence was collected corroborating the currently accepted mechanism [8,81,82,83].

### 5.3. Morita–Baylis–Hillman reaction co-catalyzed by ionic liquids

**Scheme 27:**
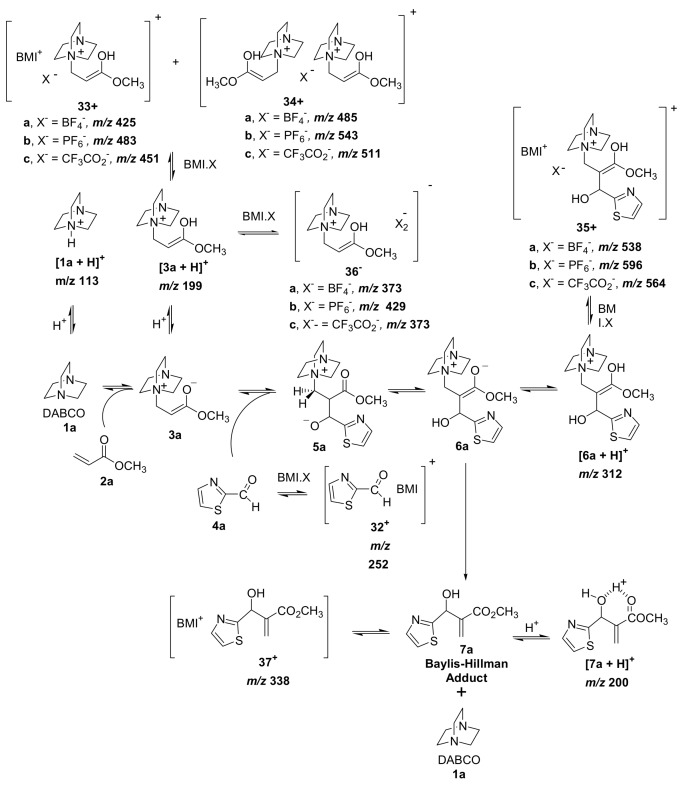
Morita–Baylis–Hillman reaction of methyl acrylate (2a) and 2-thiazolecarboxaldehyde (23) co-catalyzed by both DABCO (1a) and an ionic liquid BMI.X (X = BF_4_˜, PF_6_˜, CF_3_CO_2_˜).

In a similar manner, online monitoring of Morita–Baylis–Hillman reactions co-catalyzed by ionic liquids was applied to gently fish from solution to the gas phase supramolecular species responsible for the co-catalytic role of ionic liquids in the reaction, Scheme 27 [84]. Several supramolecular species formed by coordination of reagents and products were trapped, identified and characterized via MS analysis and MS/MS dissociations. Via competitive experiments, it was also reported the efficiency order of different co-catalysts: BMI.CF_3_CO_2_ > BMI.BF_4_ > BMI.PF_6_, which was the opposite to that observed by Afonso [85] in the liquid phase. Based on the interception of these unprecedented supramolecular species, it was proposed that 1,3-dialkylimidazolium ionic liquids function as efficient co-catalysts for the reaction by: (i) activating the aldehyde toward nucleophilic attack via BMI coordination (species **32**^+^) and (ii) by stabilizing the zwitterionic species that act as the main BH intermediates through supramolecular coordination (species **33**^+^, **34**^+^, **35**^+^ and **37**^+^).

### 5.4. Dualistic nature of Morita–Baylis–Hillman reaction probed by ESI–MS

**Scheme 28:**
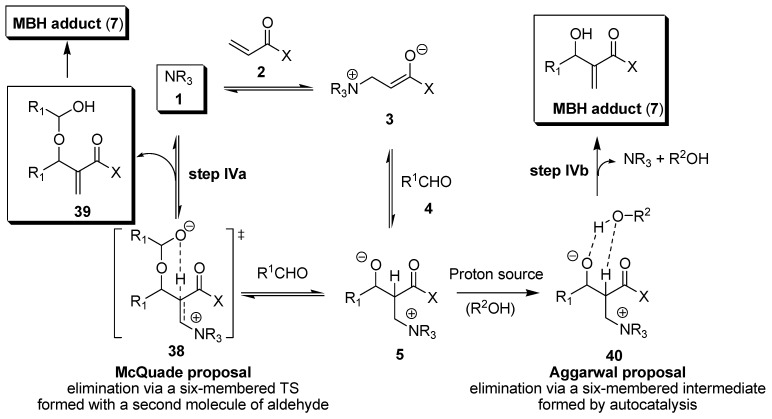
MBH reactions and the dualistic nature of the rate-determining step (RDS) elimination step.

Recently, Eberlin and coworkers employed ESI–MS(/MS) monitoring to intercept and characterize new key intermediates for the rate determining step of MBH reactions [86]. These ESI–MS data provide evidence supporting these recent suggestions based on kinetic experiments and theoretical calculations for the dualist nature of the elimination step of the MBH reaction mechanism depicted in Scheme 28. The new kinetic evidence has stimulated theoretical studies on the MBH reaction mechanism. These studies suggested that step **IV** can occur via two pathways: a) in the absence of a proton source, elimination is assisted by a second molecule of aldehyde as proposed by McQuade (Scheme 3); b) In presence of a proton source such as an alcohol, however, the elimination proceeds via intermediate **40** or a similar species, as proposed by Aggarwal (Scheme 4).

**Scheme 29:**
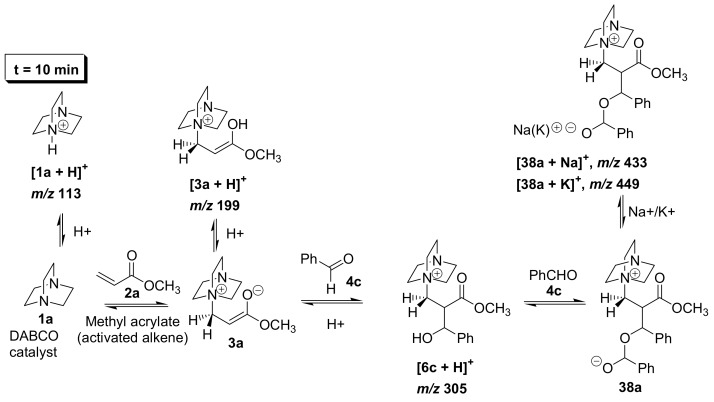
Species characterized by off-line ESI–MS(/MS) monitoring for a MBH after 10 min of reaction.

These mechanistically important new propositions about the elimination step of the MBH reaction have therefore stimulated us to perform complementary ESI–MS(/MS) investigations aiming to intercept and characterize the new MBH reaction intermediates postulated for the dualist nature of the key RDS elimination pathway. After 10 min of reaction, an aliquot of a MBH reaction solution was diluted in acetonitrile and its ESI–MS collected. Intermediate **38a** (R = Ph) proposed by McQuade [14] from the nucleophilic attack of the MBH alkoxyde to the aldehyde (Scheme 29) was intercepted as [**38a** + Na]^+^ of *m/z* 433. Considering Aggarwal’s proposal [15] for proton sources, the MBH reaction was monitored with the same experimental protocol using 1,1´-bi-2-naphthol (BINOL), ESI–MS off-line monitoring of the reaction detected a new species corresponding to [**38** + H]^+^ (R^2^OH = BINOL) at *m/z* 449. The interception and characterization of [**38** + H]^+^ agreed with Aggarwal’s proposal that a proton source participates in the elimination step by assisting the removal of the base. The “fishing” and structural characterization of these key intermediates exemplifies the complex equilibrations occurring during MBH reactions.

### 5.5. (Thio)ureas catalyzed Morita–Baylis–Hillman reaction analyzed by ESI–MS

**Figure 12:**
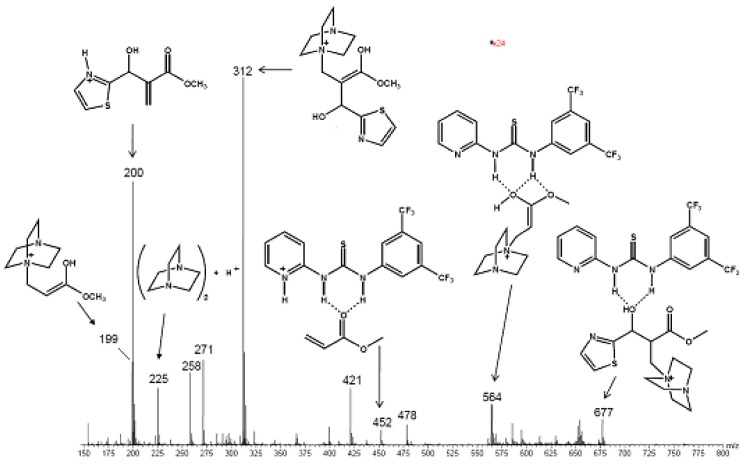
ESI(+)–MS of the MBH reaction solution co-catalyzed by a (thio)urea.

**Scheme 30:**
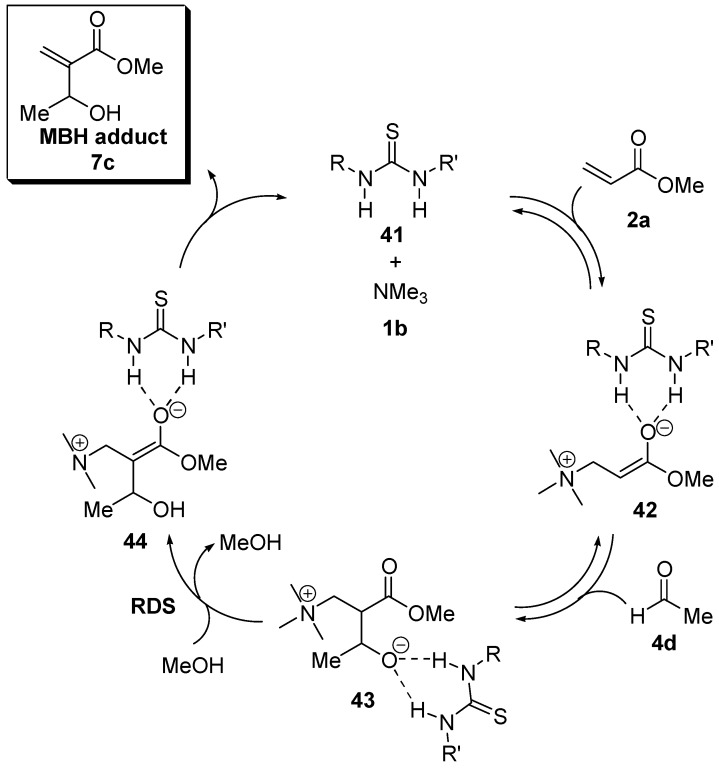
Proposal based on the ESI–MS(/MS) data for the catalytic role of (thio)urea in MBH reactions.

The catalytic role of (thio)ureas on the mechanism of the MBH reaction leading to the impressive improvements in yield, rate and enantioselectivity (see Section 4) was still unclear. Based on ESI–MS(/MS) monitoring and DFT calculations, Eberlin and coworkers proposed a new mechanism for the role of (thio)ureas on the organocatalysis of MBH reactions [87]. An MBH reaction catalyzed by (thio)urea was monitored and key intermediates were intercepted and characterized (Figure 12). The intermediates were intercepted in almost all steps of the MBH cycle proving the participation of (thio)urea as organocatalyst. These key ESI–MS(/MS) findings indicated therefore that (thio)urea participated on the RDS, that is, on the proton transfer step. DFT calculations, performed for a model MBH reaction between formaldehyde and acrolein and trimethylamine as base, either with the presence or the absence of (thio)urea, suggested that (thio)urea accelerated MBH reactions by significantly decreasing the TS energies of all reaction steps in the catalytic cycle, including therefore the rate-limiting proton transfer step. Based on the combined ESI–MS(/MS) and DFT results, a new proposal for the catalytic role of (thio)urea in the MBH reaction was presented (Scheme 30).

## 6. Conclusions

In the last years great effort has gone into the search for methods to improve the yield and shorten the reaction times, and especially the search of asymmetric inductors or catalysts for MBH reactions. Many combinations of temperature, solvent and additives such as Bronsted or Lewis acids and bases have been tested. Several catalysts including phosphines, tertiary amines, binols, amino acids, enzymes and (thio)ureas for MBH reactions have been described. Some synergistic effects were observed, and several mechanisms based on different catalysts have been proposed. The dual mechanism of MBH was also probed by online ESI–MS(/MS), as well as the role of ionic liquids as additives and the participation of the (thio)urea as organocatalysts. Although great progress in the mechanism and the discovery of different methods to improve the yields and reaction rates of this versatile reaction has been done, there is much room for catalytic asymmetric methods to further extend the scope of this useful reaction. Online purification will undoubtedly contribute to the importance of API–MS for future studies of labile and sensitive intermediates in solution, with no need for previous purification or isolation for further characterization of active species, reactive intermediates, and products. Such new MS techniques are being used to probe mechanisms of these reactions of fundamental importance and practical use by transferring the reaction intermediates directly from solution to the gas phase. The mechanistic aspects revealed can assist in the design of new catalysts for this important organic reaction.
